# Nurses’ Experiences and Factors Related to Their Attitudes Regarding Discussions with Patients and Family Members about Do-Not-Resuscitate Decisions and Life-Sustaining Treatment Withdrawal: A Hospital-Based Cross-Sectional Study

**DOI:** 10.3390/ijerph17020557

**Published:** 2020-01-15

**Authors:** Hsiao-Ting Chang, Ming-Hwai Lin, Chun-Ku Chen, Tzeng-Ji Chen, Shinn-Jang Hwang

**Affiliations:** 1 Department of Family Medicine, Taipei Veterans General Hospital, Taipei 11217, Taiwan; minghwai@gmail.com (M.-H.L.); tjchen@vghtpe.gov.tw (T.-J.C.); sjhwang@vghtpe.gov.tw (S.-J.H.); 2 School of Medicine, National Yang-Ming University, Taipei 11221, Taiwan; ckchen@vghtpe.gov.tw; 3 Department of Radiology, Taipei Veterans General Hospital, Taipei 11217, Taiwan; 4 Institute of Hospital and Health Care Administration, National Yang-Ming University, Taipei 11221, Taiwan

**Keywords:** do-not-resuscitate, end-of-life care, health care decision-making, life-sustaining treatments

## Abstract

This study aimed to evaluate nurses’ experiences and factors related to their attitudes regarding discussions of do-not-resuscitate (DNR) and withdrawal of life-sustaining treatment (LST) with patients and their families. A cross-sectional survey was conducted in a tertiary hospital in Taiwan. Nurses aged ≥ 20 years who were in charge of acute inpatient care were randomly recruited. A semi-structured questionnaire was used to evaluate participants’ experiences and attitudes regarding discussions of DNR and LST withdrawal for terminal patients. Logistic regression with adjustment for covariates was used to analyze factors related to participants’ attitudes toward discussions about DNR and LST withdrawal with patients and families in the future care of terminal patients. The participants were 132 nurses. They had significantly more discussions about DNR and LST withdrawal with patients’ families than with patients. Regression analysis showed that participants who had past experiences in actively initiating DNR discussions with patients or patients’ families were significantly more likely to discuss DNR with patients in the future care of terminal patients, but participants aged 40.0 to 60.0 years were significantly less likely to have DNR discussions than those aged 20.0 to 29.9 years. Experiences of actively initiated DNR or LST discussions with patients’ families were significantly more likely to discuss DNR with patients’ families, but those aged 40.0 to 60.0 years were also significantly less likely to have DNR discussions than those aged 20.0 to 29.9 years. Experience in actively initiating discussions about LST withdrawal with patients’ families, being male, and possessing an education level higher than university were significantly related to LST withdrawal discussions with terminal patients or their families in the future. In conclusion, there need to be more discussions about DNR and LST withdrawal with patients. To protect patients’ autonomy and their rights to make decisions about their DNR and LST, measures are needed to facilitate DNR and LST discussions with patients to ensure better end-of-life care.

## 1. Introduction

The 2000 Hospice Palliative Care Act in Taiwan was designed to protect terminally ill patients’ rights and wishes regarding their medical treatment. The act permits physicians to discuss with patients (if they have the cognitive ability to make decisions) or their families (if patients are not capable or in a comatose state) their wishes regarding do-not-resuscitate (DNR) decisions, hospice palliative care, and life-sustaining treatments (LST); only if two disease-specific specialists diagnose that a patient is in a terminal disease stage do such advance directives become effective [1]. Another law to protect patients’ rights is the 2016 Patient Right to Autonomy Act, which allows patients with the necessary decision-making capacity to make advance decisions about the complete or partial acceptance or refusal of LST and/or artificial nutrition and hydration under specific clinical conditions. This law also states that when terminating, withdrawing, or withholding LST or artificial nutrition and hydration, the medical institution or physician should provide the patient with palliative care and other appropriate measures [2]. Both acts are designed to protect patients’ right and autonomy, improve terminally ill patients’ quality of life, and permit a good death.

When treatment outcomes are insufficient for a terminally ill patient to live a meaningful life, the treatment goal may transition toward care that is comfort-oriented [3]. At that time, patients may consider DNR, which means that cardiopulmonary resuscitation will not be performed on patients in the terminal stage of incurable diseases to allow them a peaceful death. Another reason for withholding or withdrawing LST or support such as mechanical ventilation (MV), renal replacement therapy, vasopressors, tube feeding, and hydration is to reduce the suffering of patients and their families [4]. Withdrawal of MV means the removal of machines that facilitate respiration from patients who are critically or terminally ill; the aim is to avoid prolonging the dying process and to allow the patient a good death when it has become clear that he/she cannot benefit from or maintain a meaningful life by further aggressive medical treatments [4,5,6,7]. Withholding or withdrawing dialysis may also be considered in critically ill or terminal patients with acute renal failure or end-stage renal diseases [8,9,10]. 

Several studies on different aspects of withholding or withdrawal of LST in end-of-life (EOL) care have been conducted. Some have focused on legal perspectives on these practices and treatments [11,12], whereas others have examined pressures on physicians and/or families regarding withdrawal of LST [13,14]. One study conducted across 16 countries and regions in Asia that focused on healthcare professionals’ practices of withholding or withdrawal of LST found that physicians in intensive care units often withheld but seldom withdrew LST, and that their attitudes and practices differed between regions and countries [15]. There is also evidence of differences in the practice of LST withdrawal [16,17]. One study reported that extubation before the patient’s death was associated with better family satisfaction with medical care [18]. Some research has focused on factors related to withdrawal of MV [4,19], and the perceptions [7] or satisfaction [18] of patients’ families. In 2016, there were several studies on family preparation [20], communication [21,22], and support before and during LST withdrawal [20]. A descriptive study conducted in Korea, Japan, and China found that differences in the social status, moral values, religious beliefs, and economic status of each country were associated with physician attitudes toward LST withdrawal [23].

Nurses play an important role in taking care of patients at bedside, and they also spend a lot of time communicating with patients and their families. A previous questionnaire survey study in Finland reported that 46% of nurses working in the neurology, oncology, internal medicine and primary health care participated in patients’ DNR discussions [24], and another study revealed that staff nurses working in two teaching hospitals in New York believed that they should be allowed to initiate DNR discussions and were confident in this discussion [25]. A qualitative study conducted in Canada found that building capacity of the healthcare team to engage in decision-making discussions about LST with seriously ill patients and their families is needed [26]. Despite these evidences from previous studies, nurses’ experiences and factors related to their attitudes regarding discussions about DNR and LST withdrawal with patients and their families remain unclear, especially in Asian countries. Therefore, the aim of this study was to explore these two issues, in the hope to examine whether patients’ rights and autonomy to make decisions about their treatments were protected.

## 2. Materials and Methods

### 2.1. Ethical Statement

This study was approved by the institutional review board of Taipei Veterans General Hospital, Taipei, Taiwan (2017-01-004AC). Informed consent was obtained from all individuals before participation in this study.

### 2.2. Setting and Participants

This study was a cross-sectional survey study conducted in a tertiary hospital that has a well-organized hospice palliative care team comprising physicians, nurses, clinical psychotherapists, social workers, a spiritual therapist, art therapists, and a music therapist. The care team provides hospice inpatient care, hospice shared care, and hospice home care in northern Taiwan. Nurses ≥ 20 years old in charge of acute inpatient care who referred patients for discussion about DNR or withdrawal of life-sustaining treatments were randomly recruited from August through October in 2018. Nurses younger than 20 years, those mainly in charge of administrative affairs, and those whose main duty was hospice palliative care were excluded. The estimated sample size was 119 estimated by an odds ratio of 2.0 of the main outcome and a power of ≥0.9.

### 2.3. Measures

A semistructured questionnaire was developed to evaluate nurses’ experiences and attitudes regarding DNR and withdrawal of LST with patients and their families. Validity was assessed by an expert panel of two medical doctors and five palliative care nurses who all had at least 10 years of experience in clinical care. The questionnaire was then pilot-tested by 20 nurses, attending physicians, and residents. The questionnaire contained four components: questions about past experiences of DNR and LST withdrawal discussions; questions about attitudes toward discussions about DNR and LST withdrawal in the future care of terminally ill patients; open questions about withdrawal of MV; and questions on demographic information (respondents’ age, sex, education level, religious beliefs, and marital status). For the first, second, and third components, respondents’ answers to each question were recorded. The items assessing past experiences of DNR and withdrawal of LST were as follows: 1. Have you ever actively initiated DNR discussions with patients? 2. Have you ever actively initiated DNR discussions with patients’ families? 3. Have you ever actively initiated discussions on withdrawal of LST with patients? 4. Have you ever actively initiated discussions on withdrawal of LST with patients’ families? 5. Have you ever been asked to withdraw MV by terminal patients? 6. Have you ever been asked to withdraw MV by terminal patients’ families? 7. Have you ever initiated discussions on withdrawal of MV with patients after other professionals’ recommendations? 8. Have you ever initiated discussions on withdrawal of MV with patients’ families after other professionals’ recommendations? Possible responses to these questions were “no,” “yes,” or “other” (respondents were asked to provide an explanation). The items assessing attitudes to discussion of DNR and LST withdrawal were as follows: 1. Will you discuss DNR decisions with patients when taking care of terminally ill patients in the future? 2. Will you discuss DNR decisions with patients’ families when taking care of terminally ill patients in the future? 3. Will you discuss withdrawal of LST decisions with patients when taking care of terminally ill patients in the future? 4. Will you discuss withdrawal of LST decisions with patients’ families when taking care of terminally ill patients in the future? 5. What kind of LST withdrawal will you recommend to patients or their families? [The LST included (1) artificial nutrition, (2) vasopressors, (3) inotropes, (4) renal replacement therapy, (5) mechanical ventilation, (6) noninvasive positive pressure ventilation (7) antibiotics, (8) blood transfusion, and (9) others]. 6. What are your attitudes toward MV withdrawal discussions when taking care of terminally ill patients in the future: (1) I will never initiate discussions, (2) I will consider initiating discussions if the patients or their families asked, (3) I will consider initiating discussions if other professionals recommended it, (4) To decrease patients’ suffering, maintain their dignity, and help them to have a good death, I will actively initiate discussions with patients, (5) To decrease patients’ suffering, maintain their dignity, and help them to have a good death, I will actively initiate discussions with patients’ families, and (6) Other. The third component of this questionnaire constituted open questions: (1) If you will never consider initiating MV withdrawal discussions, please explain why, (2) If you will consider initiating discussions if patients or their families asked, please explain why, (3) If you will consider initiating discussions if other professionals recommended it, please explain why (Appendix A).

### 2.4. Statistical Analysis

Statistical analyses were performed using IBM SPSS version 20.0 (IBM Corporation, Armonk, NY, USA). Descriptive statistics [number (*n*) and percentage (%)] were used to analyze the categorical variables of demographic characteristics and nurses’ past experiences and attitudes toward DNR and LST withdrawal. Logistic regression analyses with adjustment for covariates including demographic characteristics (age was categorized as 20.0 to 29.9 years old, 30.0 to 39.9 years old and 40.0 to 60.0 years old; marital status was categorized as married and single; education level was categorized as university and above university; religious beliefs were categorized as yes or no) and experiences of DNR and LST withdrawal discussions were used to analyze factors related to nurses’ attitudes toward discussions of DNR and LST withdrawal with terminally ill patients and their families in the future. A two-tailed *p* value < 0.05 was considered statistically significant.

## 3. Results

### 3.1. Characteristics of Participants

One-hundred and thirty-two registered nurses were included in this study and their mean age = 37.0 ± 8.5 years. Most participants were female (*n* = 116, 87.9%), single (*n* = 75, 56.8%), and had religious beliefs (*n* = 85, 64.4%) (Table 1).

### 3.2. Participants’ Experiences and Attitudes Regarding Discussions of DNR and LST Withdrawal

One hundred and twenty eight (97.0%) participants had experiences in taking care of terminally ill patients. A total of 78 (61.9%) had experiences in actively initiating DNR discussions with patients and 91 (72.2%) had actively initiated DNR discussions with patients’ families (*p* < 0.0001); 27 (21.8%) had actively initiated discussions on LST withdrawal with patients and 49 (39.5%) had actively initiated discussions on LST withdrawal with patients’ families (*p* < 0.0001). A total of 89 (72.4%) would discuss DNR decisions with patients when caring for terminally ill patients in the future and 99 (80.5%) would discuss DNR decisions with terminally ill patients’ families in the future (*p* < 0.0001). A total of 58 (48.3%) would discuss decisions about LST withdrawal with terminally ill patients in the future and 66 (55.0%) would discuss decisions about LST withdrawal with terminally ill patients’ families in the future (*p* < 0.0001) (Table 2). The top three LST the nurses would recommend are mechanical ventilation (*n* = 85, 66.4%), renal replacement therapy (*n* = 83, 64.8%), and vasopressors (*n* = 78, 60.9%).

### 3.3. Factors Related to Attitudes toward Discussions of Do-Not-Resuscitate (DNR) Decisions for Terminally Ill Patients in the Future

Regarding factors related to attitudes toward DNR discussions, after controlling for covariates, respondents who had experiences of actively initiating DNR discussions with patients or their families were significantly more likely to have such discussions with patients [odds ratio (OR) = 3.25, 95% confidence interval (CI) = 1.01–10.50, *p* = 0.048 and OR = 5.45, 95% CI = 1.65–17.96, *p* = 0.005, respectively]; respondents who aged 40.0 to 60.0 years old were less likely to have DNR discussions with patients than respondents aged 20.0 to 29.9 years old (OR = 0.082, 95% CI = 0.01–0.55, *p* = 0.01). Respondents who had experiences of actively initiating DNR or withdrawal of LST discussions with patients’ families were more likely to have such discussions with patients’ families in the future (OR = 5.03, 95% CI = 1.31–19.34, *p* = 0.019; OR = 7.13, 95% CI = 1.12–45.54, *p* = 0.038), but those who aged 40.0 to 60.0 years old were less likely to have DNR discussions with patients’ families than respondents who were aged 20.0 to 29.9 years old (OR = 0.058, 95% CI = 0.01–0.49, *p* = 0.009) (Table 3).

### 3.4. Factors Related to Attitudes toward Discussions of Life-Sustaining Treatment (LST) Withdrawal for Terminally Ill Patients in the Future

Regarding factors related to attitudes toward discussions of LST withdrawal for terminally ill patients in the future, after controlling for covariates, respondents who had experiences of actively initiating discussions about LST withdrawal with patients’ families, were male and had an education level above university were significantly more likely to have such discussions with patients (OR = 5.55, 95% CI = 1.60–19.26, *p* = 0.007; OR = 25.50, 95% CI = 2.68–243.01, *p* = 0.005 and OR = 29.48, 95% CI = 4.19–207.32, *p* = 0.001, respectively). Respondents who had experiences of actively initiating discussions about LST withdrawal with patients’ families, were male and had an education level above university were significantly more likely to have LST withdrawal discussions with patients’ families (OR = 13.52, 95% CI = 3.25–56.23, *p* < 0.0001; OR = 25.65, 95% CI = 2.64–249.42, *p* = 0.005, and OR = 39.84, 95% CI = 3.58–443.66, *p* = 0.003, respectively) (Table 4).

Participants who said that they will discuss DNR or LST withdrawal when caring for terminally ill patients in the future mentioned the reasons of reducing patients’ suffering, maintaining patients’ dignity, and helping them to have a good death (*n* = 33).

### 3.5. Open-Ended Questions for Mechanical Ventilation Withdrawal Discussions

Six respondents responded that they will never consider initiating MV withdrawal discussions for the following reasons: they did not have the right to make decisions about another’s life, different opinions of families, afraid of legal problems, unable to detect the prognosis of patients, discussions of DNR and LST withdrawal were the responsibility of physicians, or difficulty in starting such discussions; 48 responded that they would consider initiating MV withdrawal discussions if patients or their families asked to withdraw MV to respect the patients’ autonomy or their families’ decisions (*n* = 10), and 44 would consider initiating MV withdrawal discussions if other professionals recommended it because it probably confirms that the prognosis of the patient is poor and MV might cause more suffering of the patient or his/her families (*n* = 7), and these discussions were recommended by the healthcare teams (*n* = 11).

## 4. Discussion

This study aimed to evaluate nurses’ experiences and factors related to nurses’ attitudes regarding discussions with patients and family members about DNR and LST withdrawal to evaluate whether patients’ rights and autonomy to make decisions about their treatments were protected. There were four significant findings. First, respondents were more likely to discuss DNR and LST withdrawal with patients’ families than with patients. Second, more respondents said that they will discuss DNR and LST withdrawal with patients and their families when caring for terminal patients in the future. However, they were more willing to have such discussions with patients’ families than with patients. Third, the adjusted logistic regression models showed that respondents who had experiences of actively initiating DNR discussions with patients or with patients’ families were significantly more likely to discuss DNR with patients in the future care of terminal patients, but those aged 40.0 to 60.0 years were significantly less likely to have DNR discussions than those aged 20.0 to 29.9 years. Respondents who had experiences of actively initiating DNR discussions with patients’ families were significantly more likely to discuss DNR with patients’ families in the future, but those aged 40.0 to 60.0 years were significantly less likely to have DNR discussions than those 20.0 to 29.9 years. Fourth, the adjusted logistic regression models showed that experiences of actively initiating discussions on LST withdrawal with patients’ families, being male, and possessing an education level of above university were significantly related to LST withdrawal discussions with terminal patients or their families in the future.

Previous studies have identified several factors related to physicians’ and nurses’ attitudes to DNR and LST decision discussions. Generally, physicians consider their duty to resolve patients’ health problems and save lives. People believe that patients are admitted to hospital to have their diseases treated by physicians and nurses and to regain their health, not to prepare for death. Therefore, DNR and LST discussions are avoided because death and dying are taboo topics in hospitals [26]. Concerns about legal [11,12,15] and ethical issues [13,14,15] are also related to DNR and LST discussions. In Taiwan, the Hospice Palliative Care Act regulates physician–patient discussions about patients’ wishes regarding DNR, hospice palliative care, and LST. Another law to protect patients’ rights is the Patient Right to Autonomy Act, which allows patients to make advance decisions about LST and/or artificial nutrition and hydration. The law also states that when terminating, withdrawing, or withholding LST or artificial nutrition and hydration, the medical institution or physician shall provide the patient with palliative care and other appropriate measures [2]. Despite these two acts, the present findings show that there were fewer DNR and LST discussions with patients than there were with families. Both ethical and cultural issues may be related to such discussions. Nurses face personal ethical dilemmas [13,14,15] and ethical issues in decision-making with patients and families [26,27]; they must also consider traditional cultural notions of filial piety [26,28]. Another concern is the role of anticipation in the medical context. Humans behave in an anticipatory way to adjust their behavior to prevent possible problems. Discussions of DNR and, particularly, LST withdrawal often make people think about death, the most unfavorable medical outcome [29]. For this reason, nurses may avoid discussing these issues with patients and try instead to have such discussions with families. This allows nurses to judge the reactions of families and the possible reactions of patients, and perhaps prevent “anticipation” problems.

In the present study, we found that experience of actively initiating DNR discussions with patients was significantly related to nurses’ willingness to have DNR discussions with patients in the future, but not related to willingness for discussions with family members. Experiences of actively initiating DNR discussions with patients’ families were significantly related to DNR discussions with patients and their families. Previous studies have found that DNR discussions are associated with conflicts with patients, conflicts with families, and psychological stress related to DNR decisions [30,31]. Nurses with experience in DNR decision discussions may be more confident in dealing with the conflicts of patients and their families, and with patients’ psychological distress. However, we found that nurses aged 40.0 to 60.0 years old were less likely to discuss DNR decisions with patients and family members than those aged 20.0 to 29.9 years old. This finding is in line with previous findings from the United States [25] and may reflect the emphasis on DNR discussions, palliative care, and EOL care during medical student and resident training for younger physicians and nurses, and the legislation in the Hospice Palliative Care Act and Patient Right to Autonomy Act.

Experience of actively initiating discussions on LST withdrawal with patients’ families was significantly related to LST decision discussions in this study. Withdrawing treatments is difficult and emotional for physicians and nurses, patients, and families [24,32]. Pressures on discussions about LST withdrawal may arise from patient prognosis, physician factors, nurse factors, concerns from patients’ families, social factors, and economic factors [14]. During the decision-making process, listening to patients and their families with empathy, discussing time-limited treatments or trials, maintaining provision of comfort-oriented care for patients, supporting family members to meet their needs, and engaging in constant empathic communication may help [20,22,24]. One study that used a web-based survey of anesthesiologists’ attitudes toward EOL issues in intensive care in Italy reported that 58% of discussions about LST withdrawal or withholding resulted in decisions, but that 70% of respondents’ intensive care units did not have associative supportive or palliative care; a factor possibly related to physicians’ and nurses’ reluctance to discuss LST withdrawal [33]. In our study, respondents expressed the difficulty of starting treatment-related discussions, and that they were unable to detect the prognosis of patients which suggest that education and practice are needed to build nurses’ confidence and knowledge in discussing DNR and LST withdrawal issues with patients and families. In the current study, we also found that male nurses were significantly more likely to discuss LST withdrawal with patients or their families. The reasons for the gender difference still need further evaluation.

This study had some limitations. First, as this was a cross-sectional study, causal relationships could not be examined. Second, the study was conducted in a tertiary hospital with a well-organized hospice palliative care team that included physicians, nurses, psychotherapists, and other types of therapists, and social workers, that provide hospice care. Therefore, the results may only be applicable to other hospitals of a similar level with similar facilities. Third, we did not measure years of experience for physicians and nurses. However, we collected data on experiences and attitudes from nurses who were in charge of acute inpatient care; thus, the results should be representative for this group of nurses in similar-level hospitals. Fourth, the results of logistic regression of factors related to withdrawal of LST for terminally ill patients in the future showed significantly high but wide 95% CI of OR for ‘male’ and ‘above university education level’, these two results might be related to low prevalence of these two variables in our respondents. However, these two findings should not be neglected and need to be interpreted carefully and further evaluations are needed [34].

## 5. Conclusions

Nurses had more discussions about DNR and LST withdrawal with patients’ families than with patients. The respondents were more willing to have such discussions with patients’ families than with patients in the future care of terminal patients. Nurses who had past experiences in actively initiated DNR discussions with patients or patients’ families were significantly more likely to discuss DNR with patients in the future care of terminal patients, but participants aged 40.0 to 60.0 years were significantly less likely to have DNR discussions than those aged 20.0 to 29.9 years. Nurses with experiences of actively initiating DNR or LST discussions with patients’ families were significantly more likely to discuss DNR with patients’ families, but those aged 40.0 to 60.0 years were also significantly less likely to have DNR discussions than those aged 20.0 to 29.9 years. The experience of actively initiating discussions regarding LST withdrawal with patients’ families, being male, and possessing an education level of above university were significantly related to LST withdrawal discussions with terminal patients or their families in the future. To protect patients’ autonomy and their rights to make decisions about their DNR and LST, measures to facilitate DNR and LST discussions with patients should be implemented.

## Figures and Tables

**Table 1:** Demographic characteristics of participants.

Characteristics	*n*	%
Sex		
Female	116	87.9
Education level		
University	113	85.6
Above university	19	14.4
Marital status		
Single	75	56.8
Married	57	43.2
Religious belief		
None	47	35.6
Buddhism	39	29.5
Taoism	21	15.9
General folk belief	20	15.2
Christian	4	3.0
Others	1	0.8

**Table 2:** Experiences and attitudes regarding do-not-resuscitate (DNR) and life-sustaining treatment (LST) withdrawal discussions.

Items	No		Yes		*p* Value	Missing	
	no.	%	no.	%		no.	%
Had you ever actively initiated DNR discussions with patients?	48	38.1	78	61.9	<0.0001	2	1.6
Had you ever actively initiated DNR discussions with patients’ families?	35	27.8	91	72.2		2	1.6
Had you ever actively initiated discussions on withdrawal of LST with patients?	97	78.2	27	21.8	<0.0001	4	3.1
Had you ever actively initiated discussions on withdrawal of LST with patients’ families?	75	60.5	49	39.5		4	3.1
Will you discuss DNR decisions with patients when taking care of terminally ill patients in the future?	34	27.6	89	62.4	<0.0001	5	3.9
Will you discuss DNR decisions with patients’ families when taking care of terminally ill patients in the future?	24	19.5	99	80.5		5	3.9
Will you discuss withdrawal of LST decisions with patients when taking care of terminally ill patients in the future?	62	51.7	58	48.3	<0.0001	8	6.3
Will you discuss withdrawal of LST decisions with patients’ families when taking care of terminally ill patients in the future?	54	45.0	66	55.0		8	6.3

**Table 3:** Factors related to attitudes toward discussions of do-not-resuscitate (DNR) decisions for terminally ill patients in the future.

Variable	Discuss with Patients			Discuss with Patients’ Families		
	OR	95% CI	*p* Value	OR	95% CI	*p* Value
Experiences of actively initiating DNR discussions with patients						
No	Reference			Reference		
Yes	3.25	1.01–10.50	0.048	2.62	0.64–10.61	0.180
Experiences of actively initiating DNR discussions with patients’ families						
No	Reference			Reference		
Yes	5.45	1.65–17.96	0.005	5.03	1.31–19.34	0.019
Experiences of actively initiating discussions on life-sustaining treatment withdrawal with patients						
No	Reference			Reference		
Yes	0.99	0.14–6.92	0.99	0.76	0.05–10.98	0.842
Experiences of actively initiating discussions on life-sustaining treatment withdrawal with patients’ families						
No	Reference			Reference		
Yes	2.44	0.64–9.37	0.193	7.13	1.12–45.54	0.038
Age (Year)						
20.0~29.9	Reference			Reference		
30.0~39.9	0.29	0.06–1.41	0.126	0.24	0.04–1.52	0.130
40.0~60.0	0.08	0.01–0.55	0.01	0.06	0.01–0.49	0.009
Sex						
Female	Reference			Reference		
Male	3.07	0.32–29.74	0.333	0.68	0.07–6.93	0.742
Marital status						
Married	Reference			Reference		
Single	0.61	0.18–2.11	0.433	0.71	0.18–2.80	0.626
Education level						
University	Reference			Reference		
Above university	6.88	0.99–48.10	0.052	5.16	0.51–51.93	0.163
Religious belief						
No	Reference			Reference		
Yes	0.87	0.27–2.78	0.818	1.19	0.34–4.19	0.791
Cox & Snell R-square	0.316			0.289		

OR: odds ratio; CI: confidence interval.

**Table 4:** Factors related to attitudes toward discussions of life-sustaining treatment (LST) withdrawal for terminally ill patients in the future.

Variable	Discuss with Patients			Discuss with Patients’ Families		
	OR	95% CI	*p* Value	OR	95% CI	*p* Value
Experiences of actively initiating do-not-resuscitate discussions with patients						
No	Reference			Reference		
Yes	0.80	0.27–2.41	0.692	0.88	0.29–2.71	0.824
Experiences of actively initiating do-not-resuscitate discussions with patients’ families						
No	Reference			Reference		
Yes	1.98	0.62–6.36	0.250	1.60	0.50–5.17	0.433
Experiences of actively initiating discussions on LST withdrawal with patients						
No	Reference			Reference		
Yes	0.89	0.22–3.65	0.876	0.52	0.10–2.58	0.422
Experiences of actively initiating discussions on LST withdrawal with patients’ families						
No	Reference			Reference		
Yes	5.55	1.60–19.26	0.007	13.52	3.25–56.23	<0.0001
Age (Year)						
20.0~29.9	Reference			Reference		
30.0~39.9	1.31	0.38–4.55	0.672	1.59	0.43–5.80	0.486
40.0~60.0	0.19	0.03–1.06	0.058	0.29	0.05–1.64	0.161
Sex						
Female	Reference			Reference		
Male	25.50	2.68–243.01	0.005	25.65	2.64–249.42	0.005
Marital status						
Married	Reference			Reference		
Single	0.42	0.13–1.41	0.144	0.44	0.13–1.47	0.183
Education level						
University	Reference			Reference		
Above university	29.48	4.19–207.32	0.001	39.84	3.58–443.66	0.003
Religious belief						
No	Reference			Reference		
Yes	1.96	0.70–5.47	0.199	2.14	0.73–6.25	0.164
Cox & Snell R-square	0.340			0.370		

OR: odds ratio; CI: confidence interval.

## Appendix A

**Table A1:** Questionnaire Regarding Nurses’ Experiences and Attitudes toward Discussions on Do-Not-Resuscitate (DNR) Decisions and Life-Sustaining Treatment (LST) Withdrawal.

**Component 1. Past Experiences of DNR and Withdrawal of LST Discussions**
Have you ever actively initiated DNR discussions with patients? Have you ever actively initiated DNR discussions with patients’ families? Have you ever actively initiated discussions on withdrawal of LST with patients? Have you ever actively initiated discussions on withdrawal of LST with patients’ families? Have you ever been asked to withdraw mechanical ventilation by terminal patients? Have you ever been asked to withdraw mechanical ventilation by terminal patients’ families? Have you ever initiated discussions on withdrawal of mechanical ventilation with patients after other professionals’ recommendations? Have you ever initiated discussions on withdrawal of mechanical ventilation with patients’ families after other professionals’ recommendations?
**Component 2. Attitudes to Discussing DNR and Withdrawal of LST in the Future Care of Terminally Ill Patients**
Will you discuss DNR decisions with patients when taking care of terminally ill patients in the future? Will you discuss DNR decisions with patients’ families when taking care of terminally ill patients in the future? Will you discuss withdrawal of LST decisions with patients when taking care of terminally ill patients in the future? Will you discuss withdrawal of LST decisions with patients’ families when taking care of terminally ill patients in the future? What kind of LST withdrawal will you recommend to patients or their families? (1) artificial nutrition, (2) vasopressors, (3) inotropes, (4) dialysis, (5) mechanical ventilation (MV), (6) non-invasive positive pressure ventilation (7) antibiotics, (8) blood transfusion, and (9) others. What are your attitudes toward MV withdrawal discussions when taking care of terminally ill patients in the future: (1) I will never initiate discussions. (2) I will consider initiating discussions if the patients or their families asked. (3) I will consider initiating discussions if other professionals recommended it. (4) To decrease patients’ suffering, maintain their dignity, and help them to have a good death, I would actively initiate discussions with patients (5) To decrease patients’ suffering, maintain their dignity, and help them to have a good death, I would actively initiate discussions with patients’ families (6) Others.
**Component 3. Other Open-Ended Questions**
If you will never consider initiating MV withdrawal discussions, please explain why. If you will consider initiating MV withdrawal discussions if patients or their families asked, please explain why. If you will consider initiating MV withdrawal discussions if other professionals recommended it, please explain why.
**Component 4. Demographic Information**
Sex, age, education level, religious beliefs, and marital status
